# Statistical FT-IR Spectroscopy for the Characterization of 17 Vegetable Oils

**DOI:** 10.3390/molecules27103190

**Published:** 2022-05-17

**Authors:** Meta Kokalj Ladan, Nina Kočevar Glavač

**Affiliations:** Faculty of Pharmacy, University of Ljubljana, 1000 Ljubljana, Slovenia; nina.kocevar.glavac@ffa.uni-lj.si

**Keywords:** vegetable fatty oils, chemometrics, FT-IR, quality control

## Abstract

Vegetable oils have been utilized for centuries in the food, cosmetic, and pharmaceutical industries, and they contribute beneficially to overall human health, to active skincare, and to effective treatments. Monitoring of the vegetable oils is carried out by the methods described in the European Pharmacopeia, which is time-consuming, has poor repeatability, and involves the use of toxic organic chemicals and expensive laboratory equipment. Many successful studies using IR spectroscopy have been carried out for the detection of geographical origin and adulteration as well as quantification of oxidation parameters. The aim of our research was to explore FT-IR spectroscopy for assessing the quality parameters and fatty acid composition of cranberry, elderberry, borage, blackcurrant, raspberry, black mustard, walnut, sea buckthorn, evening primrose, rosehip, chia, perilla, black cumin, sacha inchi, kiwi, hemp, and linseed oil. Very good models were obtained for the α-linolenic acid and linoleic acid contents, with *R*^2^ = 1.00; *R_v_*^2^ values of 0.98, 0.92, 0.89, and 0.84 were obtained for iodine value prediction, stearic acid content, palmitic acid content, and unsaponifiable matter content, respectively. However, we were not able to obtain good models for all parameters, and the use of the same process for variable selection was found to be not suitable for all cases.

## 1. Introduction

Vegetable oils have been utilized for centuries for a wide variety of applications, ranging from home to industrial uses. In the food, cosmetic, and pharmaceutical industries, they have been increasingly recognized as bioactive substances that contribute beneficially to overall body health, to active skincare, and to effective treatments [1,2,3,4].

Chemically, vegetable oils are mixtures of triglycerides, unsaponifiable compounds, and some other types of lipids, such as waxes and phospholipids. Triglycerides are esters of glycerol and fatty acids, which account for up to 99% of the oil content, while the unsaponifiable compound content generally ranges from 0.5% to 2%. Fatty acids are divided into saturated (e.g., lauric, myristic, palmitic, stearic), and mono- (oleic) and polyunsaturated (linolenic, linoleic). The most common unsaponifiable compounds are terpenic compounds (phytol, squalene, triterpene alcohols, phytosterols, carotenoids, vitamin E), phenolic compounds (flavonoids, ferulic acid), and gamma oryzanol [3].

The total fatty acids, unsaturated fatty acids, and oxidation-prone unsaponifiable compounds themselves are the most critical elements of the oil’s chemical composition, as their deterioration can not only lower health benefits but also lead to undesired or even toxic effects, e.g., through the formation of reactive oxygen species and trans fatty acids [5,6,7].

In terms of dietary, cosmetic, and pharmaceutical uses of vegetable oils, it is, therefore, of extreme importance to regularly monitor vegetable oil quality. For a comprehensive quality evaluation of vegetable oils, a combination of different methods must be used. According to the European Pharmacopoeia, which provides standards for the quality control of medicines in the European Union, the following parameters are used for the evaluation of vegetable oils: composition of fatty acids, peroxide value, iodine value, acid value, hydroxyl value, ester value, saponification value, and unsaponifiable matter [8]. The major general drawbacks of the methods used for evaluating these parameters include the time-consuming work, the analyst’s error in manual titration and determination of the endpoint (e.g., development or disappearance of the color), poor repeatability, and the use of toxic organic chemicals and expensive laboratory equipment [9,10].

Infrared spectroscopy is a widely used method for qualitative and quantitative analysis. It is fast, easy to use, and environmentally friendly. It is frequently coupled with chemometrical methods that allow appropriate spectral handling and models development, and improves the extraction of relevant spectral information [11,12,13,14]. An important step in data handling is preprocessing of the spectra. The main goals of the preprocessing are noise elimination and increasing sensitivity. The most commonly used techniques for noise elimination are normalization, standard normal variate (SNV), multiplicative scatter correction (MSC), and other smoothing techniques. For increasing sensitivity, the most used technique is derivation. It is very important to find a good balance between the smoothing and increasing sensitivity techniques in each application [15,16]. The gold standard of the multivariate quantitative model-building technique is the partial least squares (PLS) method [16,17]. The method builds a model using independent variables with different weights to predict a dependent variable of interest. A very important step in model building is appropriate validation with an independent validation set of samples [18].

The use of infrared spectroscopy for vegetable oil analysis is not new [19]. Many successful studies for the detection of geographical origin and adulteration as well as quantification of oxidation parameters have been conducted [20,21,22,23,24,25,26,27,28,29,30,31]. Most studies are performed on olive oil and edible oils used for frying [23,27,28,29,30,31,32]. Additionally, the number of samples is usually low, or only one type of oil is used. Samples are usually prepared by heating or oxidizing the oils to different degrees [22,24,25,33]. However, only a few studies have been conducted for a larger set of different oils [34] and for many different parameters [27] and never for the types of oils used in this study.

The aim of our research was to explore FT-IR spectroscopy as an alternative method for assessing the quality parameters prescribed by the European Pharmacopoeia (acid, ester, hydroxyl, iodine, peroxide, saponification value, and unsaponifiable matter) and fatty acid composition (palmitic, linoleic, α-linolenic, oleic, elaidic, and stearic acid) of cranberry, elderberry, borage, blackcurrant, raspberry, black mustard, walnut, sea buckthorn, evening primrose, rosehip, chia, perilla, black cumin, sacha inchi, kiwi, hemp, and linseed oil.

## 2. Results

The purpose of our research was to explore IR spectroscopy and multivariate modeling for obtaining predictive models for parameters used to assess the quality of vegetable oils. The results are presented by comparing different spectral acquisition techniques and data processing techniques and for 13 different dependent variables. First, the results of comparing spectral and random data are discussed, and then ATR and transmissive techniques are compared. Additionally, three resolutions of 2 cm^−1^, 4 cm^−1^, and 8 cm^−1^, the use of averaged or all three spectra separately, and spectral data reduction techniques were used. These results apply similarly to all dependent variables. 

### 2.1. Spectral and Random Data

Infrared spectra contain a large number of spectral points, variables. With these variables, predictive models are built using statistical data processing methods. Due to the large number of these variables, there is a risk that a good model is obtained due to chance and not sample information. We wanted to check in practice and in our actual case that the obtained models are the result of important information in the spectrum and not incorrect data processing. Therefore, we designed a set of variables with random values to use instead of infrared spectra, and with them, we performed the same model building process as with the spectral data. We checked the statistical parameters of the models obtained with these random datasets and confirmed the assumption that with such a large set of independent variables, a model with good statistical parameters for the learning calibration set can be built, but the parameters for the validation set do not exceed the values that are obtained due to mere chance.

The results are shown in Table 1. Thus, *R_c_*^2^ values obtained for the models built on random data are as high as 1.0, while *R_v_*^2^ exceeds 0.5 only in one case with a value of 0.55 (Table 1). Models built using the spectral data show that both statistical parameters *R_c_*^2^ and *R_v_*^2^ are as high as 1.0. In all further discussion of the results, only models built using real spectral data are discussed. Table 1 also shows that the percent of models with a good *R_c_*^2^ value is higher for the case of random data. This is probably because the data in the spectral dataset are not entirely independent, and some contain information that is similar to that in the other datasets; therefore, it is easier to fit the data in the random dataset to the dependent variable.

### 2.2. ATR versus Transmissive Spectra

Representative ATR and transmissive spectra are shown in Figures S1 and S2, respectively. In our experiments, more good models (with both *R*^2^ > 0.5) were obtained using the transmissive spectra measuring technique. This is shown in Table 2. For most of the dependent variables, good models with both spectrum recording modes were built; the recorded transmissive spectra gave models with *R*^2^ above 0.9 only for predicting stearic acid content, whereas ATR spectra did not produce such good results. For the ATR spectra recording technique, the light penetrates into the sample by only a few micrometers and, therefore, is not sensitive enough for some purposes. However, the ATR recording method is easier to implement and requires less sample preparation.

### 2.3. Resolution

As shown in Table 2, most models using a resolution of 8 cm^−1^ gave *R_c_*^2^ and *R_v_*^2^ values of larger than 0.9 and 0.5, respectively. However, good models were also obtained with resolutions of 2 cm^−1^ and 4 cm^−1^. A resolution of 8 cm^−1^ also has other advantages: there are a smaller number of data points, and the spectra are recorded faster.

### 2.4. Averaged Spectra

We compared models by either averaging over three spectra or using three spectra separately, and the results are shown in Table 2. Averaging the spectra lowers the noise, especially due to sample preparation and spectral acquisition. However, three separate spectra may give additional information and are more similar to the use of only one spectral recording, which enables faster sample analysis. Both methods gave models with *R_c_*^2^ and *R_v_*^2^ larger than 0.9 and 0.5, respectively. Overall, using all spectra gave better results.

### 2.5. Data Selection

Spectral data contain a large number of data points, of which not all are important for building a good model. Moreover, they make the predictive model worse [18]. Therefore, it is beneficial to select some spectral variables prior to multivariate analysis. In our work, three techniques were compared: First, the standard deviation among samples for each wavenumber was calculated, choosing those with the largest deviation, as these variables contain the most differentiating information (STD). The second technique involved calculating the correlation coefficients for wavenumbers with dependent variables and using those with the highest correlation (CORR). The third technique used parts of the spectra that are characteristic of important chemical bonds (CHEM). The results are shown in Table 2. All three methods gave models with *R_c_*^2^ and *R_v_*^2^ larger than 0.9 and 0.5, respectively. Overall, using parts of the spectra that are characteristic of important chemical bonds gave slightly better results.

### 2.6. Preprocessing Technique

The purpose of preprocessing spectral data is to remove noise and emphasize important information. Different pretreatment techniques were used. Table 1 shows the percent of models with good *R_v_*^2^ and *R_c_*^2^ obtained with each pretreatment technique. For the case of all data, within each type of pretreatment, using a derivative gave worse results. We also notice that with normalization and SNV normalization, the number of very good models with *R_v_*^2^ higher than 0.99 and 0.95 increased. As the presence of noise is greatly influenced by the resolution, we expect more noise at higher resolution; therefore, the results obtained by using individual resolutions are also presented in Table S1. If we look at the results based on resolution, we notice that the deterioration with the derivative was obvious at a resolution of 2 cm^−1^; however, at a resolution of 8 cm^−1^, this connection was lost. It can be observed that normalization and SNV normalization improved the percent of good models in all three resolutions; with these two pretreatments for the case of a resolution of 8 cm^−1^, the first derivative slightly improved the results. Overall, the best results were obtained with a resolution of 8 cm^−1^. With this resolution, there were many preprocessing techniques that worked well; therefore, it is difficult to name only one.

### 2.7. Dependent Variables

Models with both *R*^2^ above 0.9 were obtained for linoleic acid, α-linolenic acid, stearic acid content, and iodine value. Models with both *R*^2^ above 0.5 were obtained for palmitic acid, oleic acid, elaidic acid, and unsaponifiable matter content. Models for the acid value, saponification value, ester value, hydroxyl value, and peroxide value did not have both *R*^2^ above 0.5. Table S1 shows the results obtained for the models for each predictive variable using different preprocessing results. In general, the best preprocessing techniques were normalized spectra and SNV spectra. However, WA, raw spectra, and first derivatives produced good results, and worse results were obtained using detailed wavelet coefficients and their derivatives and second derivatives.

## 3. Discussion

**Figure 1:**
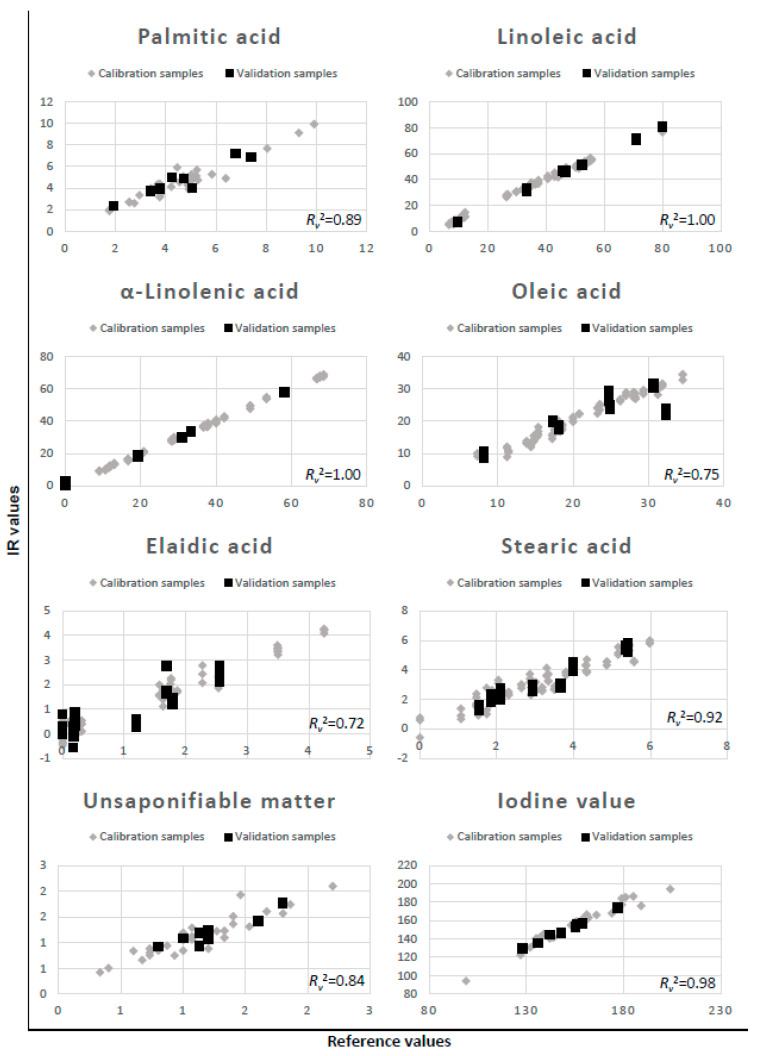
Best results obtained for palmitic, linoleic, α-linolenic, oleic, elaidic, stearic acid, unsaponifiable matter content, and iodine value models; *R_v_*^2^ values are also given.

The best models for each dependent variable are presented in Table 3 and Figure 1. In Table 3, all parameters of the three models with the highest *R_v_*^2^ and lowest RMSEP were given for each of the dependent variables. Models with good *R*^2^ values also had low RMSEP and comparable RMSEC and RMSEP. If RMSEC is much larger compared to RMSEP, this indicates overfitting of a model to the calibration set; this would also show as a high *R_c_*^2^ value. If both RMS are high compared to the calibration range, this shows that a good model cannot be made on a given set of independent variables.

For both the α-linolenic acid and linoleic acid models, *R*^2^ of 1.00 was obtained. In the literature models reviewed for these two fatty acids, the content percent was built by Mahboubifar et al. using four types of edible oils and observing the compositional change during heating; for linolenic and linoleic acid, *R_v_*^2^ was 0.94 and 0.99, respectively [27]. In this reference, models for palmitic acid, stearic acid, and oleic acid were also built, obtaining *R_v_*^2^ values of 0.99, 0.97, and 0.98, respectively. In our study, the highest *R_v_*^2^ values obtained for these fatty acid contents were 0.89, 0.92, and 0.75, respectively.

The highest *R_v_*^2^ value obtained for iodine value prediction was 0.98. Similarly, other authors have made very good models for iodine value prediction [25,34]. Dyminska et al. analyzed 13 different oils (sunflower, avocado, hemp, high-linolenic flax, low-linolenic flax, safflower, walnut, roasted sesame, rice, corn, rapeseed, pumpkin seed, hazel) and obtained a model based on IR spectral data with *R_v_*^2^ = 0.988. This study used a similarly heterogenic set of different oil types but with a smaller number of samples [34]. Additionally, Sanchez et al. analyzed the iodine value in hydrogenated soybean oil using FTIR-ATR and built a model for iodine value prediction with *R_v_*^2^ = 0.987. However, the purpose of this study was very different to ours. Only one type of oil was used, and the aim was to monitor changes in the iodine value during a very specific process: hydrogenation [25]. In our study, the aim was to control the quality of different types of specific oils not subjected to any deterioration process.

Some variables had very low *R_v_*^2^ values. Models for the peroxide value had the highest *R_v_*^2^ value of 0.49. In the reviewed literature, models for peroxide values have *R_v_*^2^ values ranging between 0.701 and 0.997. Good models for the peroxide value are mostly obtained for a small number of oil types studied, mostly single plant sources, during oxidation processes such as heating or frying [22,23,26,27]. Molecules containing -OH groups overlap with the hydroperoxide band, which interferes with the determination of the peroxide value. Such molecules are mainly alcohols, phytosterols, mono- and diglycerides, free fatty acids, and water [23].

We were not able to obtain a good model for the acid value, saponification value, ester value, and hydroxyl value with our methods. In the reviewed literature, we found that Mahboubifar et al. built a model for acid value prediction with *R_v_*^2^ 0.86 [27]. Similar to the peroxide value, the aim of this study was to monitor the variation in the composition of oil during heating. Bendini et al. built a good model for predicting the acid value for olive oils with *R_v_*^2^ 0.955; compared to our study, their sample set was composed of only one plant-type oil [23].

In our study, the same process for variable selection was used for all dependent variables. The results showed that this is not suitable for all of them. For the peroxide value, acid value, saponification value, ester value, and hydroxyl value, models can be improved by finding and selecting even more specific parts of the spectra, excluding irrelevant data for these dependent variables.

In Table 4, the FT-IR method is compared to reference methods for vegetable oil characterization. In the FT-IR method, the most time-consuming and difficult part is the method development and validation part. Once the method is developed for a given application, the analysis is fast and easily carried out with little chance of human error. In titration methods, there are many toxic chemicals used and laboratory skills are important for the accuracy and repeatability of analysis. Gas chromatography for fatty acid quantification requires appropriate sample preparation, which again uses toxic organic chemicals. Gas chromatography itself can be automated; however, the analysis is long.

## 4. Materials and Methods

### 4.1. Samples

Thirty-seven commercially available vegetable oils used as cosmetic ingredients and as food or food supplements were collected. Among these oils, there were 18 different types of oils and one to four samples for each type of oil. Each type of oil had one or two representatives in the calibration dataset; if there were three or more samples of the same type, the rest were put into the validation set. Validation samples were chosen randomly, excluding minimal or maximal values for each dependent variable where possible. The samples, manufacturer, and type of dataset are presented in Table 5.

### 4.2. Chemical Characterization of Vegetable Oils

The acid value, saponification value, ester value, hydroxyl value (method A), iodine value (method B), peroxide value (method B), and unsaponifiable matter were determined by the procedures described in European Pharmacopoeia 8.0, sections 2.5.1–2.5.7.

### 4.3. GC–MS Analysis of Fatty Acid Composition

First, the transesterification of fatty acids was carried out to make the constituents volatile for GC–MS analysis. The sample was injected to the GC column for separation of the constituents. They were detected by an MS detector and the identification was carried out by reference compounds and MS spectral libraries.

In situ transesterification was carried out for 10 mg of samples, adding 10 μL of dichloromethane and 200 μL of 0.5 M NaOH in methanol. The mixture was then heated in a water bath at 90 °C for 10 min. After cooling, 200 μL of 14% BF_3_ in methanol was added, and the mixture was heated again at 90 °C for 10 min. Then, 200 μL of demineralized water and 1 mL of hexane were added and shaken intensively for 1 min for extraction. The hexane phase was analyzed by GC–MS. A gas chromatograph (GCMS-QP2010 Ultra; Shimadzu, Kyoto, Japan) was used to analyze the material. A capillary column was used (Rtx-1 F&F; 30 m × 0.25 mm i.d.; film thickness, 0.25 μm; Restek, Bellefonte, PA, USA). The temperature program began at 160 °C and the temperature was then raised to 250 °C at 3 °C/min. The injection temperature was 250 °C, the temperature of the ion source was 200 °C, and the temperature of the interface was 280 °C. The injection volume was 1 µL, the split ratio was 1:100, the carrier gas was He, and the flow linear velocity was 1 mL/min. The mass spectrometry conditions included electron impact mode at an ionization voltage of 70 eV, with the total ion current recorded, with a scan range from 35 *m*/*z* to 500 *m*/*z* at a frequency of 5 Hz. The detector voltage was 1 kV. The total analysis time was 30 min. The compounds were identified by comparing their mass spectra and retention indices to the spectra and retention indices of the reference compounds obtained from standard Supelco F.A.M.E. MIX, C4-C24 (Sigma-Aldrich, Steinheim, Germany) and to the spectra and retention indices obtained from the Flavors and Fragrances of Natural and Synthetic Compounds spectral library (FFNSC3) and National Institute of Standards and Technology spectral library (NIST11). Concentrations were calculated as relative peak areas, and fatty acid contents are given in %.

### 4.4. Recording of the IR Spectra

#### 4.4.1. ATR Spectra

ATR FT-IR spectra were collected using a diamond attenuated total reflection (ATR) accessory from Dura SamplIR Technologies coupled to a Nicolet Instrument Co spectrometer using a DTGS detector. The spectrometer was linked to a computer equipped with Omnic E.S.P. 5.2 software to allow for the automated collection of IR spectra.

Each spectrum was collected as an average of 50 scans between 500 cm^−1^ and 4000 cm^−1^. Spectra were collected at three different resolutions: 2 cm^−1^, 4 cm^−1^, and 8 cm^−1^. Three spectra at each resolution were collected for each sample. A new background was collected at each resolution before the three measurements for one sample because of the CO_2_ peak. For data analysis and model building, all three spectra obtained for one sample were used separately or averaged.

#### 4.4.2. Transmissive Spectra

Transmissive FT-IR spectra were collected using a SpectraTech extension for NaCl plates coupled to a Nicolet Instrument Co spectrometer using a DTGS detector. The spectrometer was linked to a computer equipped with Omnic E.S.P. 5.2 software to allow for the automated collection of IR spectra.

Vegetable oils were diluted in dichloromethane vol/vol = 1/10, and one droplet was placed onto a NaCl plate (11 × 30 × 7 mm) and left alone for dichloromethane to evaporate. The NaCl plate was completely cleaned between different samples with saturated NaCl ethanol solution and only wiped between three consecutive measurements of the same sample. Each spectrum was collected as an average of 50 scans between 600 cm^−1^ and 4000 cm^−1^. Spectra were collected at three different resolutions: 2 cm^−1^ (R2), 4 cm^−1^ (R4), and 8 cm^−1^ (R8). Three spectra at each resolution were collected for each sample. A new background was collected at each resolution before the three measurements for one sample because of the CO_2_ peak. For data analysis and model building, all three spectra obtained for one sample were used separately or averaged.

### 4.5. Data Analysis

After collecting the infrared spectra, they were first preprocessed with different techniques; in the next step, the important variables were extracted by different methods. Models were then built using partial least squares regression (PLS). In the last step, the validation with an independent set of samples was carried out and parameters to assess the quality of the models were calculated.

Spectral data analyses were carried out using Octave 5.1.0. A total of 7488 models were built for all 13 predictive variables using all possible combinations of the 5 parameters (spectra measurement technique, resolution, separate or averaged spectra, predictive variable selection, and spectra preprocessing). Spectra were measured using ATR and the transmission method. Models were built using three separate spectra for each sample and compared to models built using averaged spectra for each sample. Samples were divided into calibration and validation sets. In cases where three spectra for the same sample were included in the analysis, all three spectra were assigned to the same for the two sets. Spectra were collected at three different resolutions. Different spectra preprocessing techniques were used for all spectra: raw spectra, first and second derivative, normalization (NOR), first and second derivative of normalization, standard normal variate (SNV), first and second derivative of SNV, Haar wavelet transform, and the first and second derivative of data obtained with the Haar wavelet transform. The wavelet transform yields two datasets: the approximate coefficients (WA) and the detailed coefficients (WD), both of which were compared. As there are many variables in the IR spectrum and many statistical processes, we also used random data, which were treated as one of the preprocessing techniques. In this case, the matrix data were filled with random numbers instead of spectral data. The aim was to check that it is not possible to obtain good models with random data.

Spectra contain a large number of variables; different methods were used for lowering the number of these variables to approximately 40% for spectral data before applying the partial least squares (PLS) method. In the first case (CORR), these variables were selected using the Pearson correlation, where 40% of all spectral variables were chosen based on the largest absolute correlation with the dependent variable for which the model was built. Another technique (CHEM) was to first lower the number of spectral variables, taking in the parts of the spectra where absorption peaks for important chemical bonds exist; the selected spectral ranges were 600–1500 cm^−1^, 1600–1800 cm^−1^, 2850–3050 cm^−1^, and 3400–3500 cm^−1^. The third technique (STD) used for lowering the number of variables involved selecting 40% of spectral variables based on the largest standard deviation. The hypothesis was that where spectra change most among samples, there is important information available for differentiation.

To obtain the most appropriate number of latent factors used in PLS, the leave-one-out method was used for the calibration set to test for the 1–20 latent factors used.

The quality of the models was assessed using the correlation *R*^2^ of the calibration (*R_c_*^2^) and validation (*R_v_*^2^) sets of samples and the root-mean-square error of calibration (RMSEC), root-mean-square error of validation (RMSEV), and root-mean-square error of prediction (RMSEP).

## 5. Conclusions

The methods used give good models for predicting the content of the fatty acids studied, while good models for predicting pharmacopoeia chemical values are obtained only for iodine value and unsaponifiable matter. For some variables, such as the linoleic and α-linolenic acid content and iodine number, a large number of good models are obtained. In this case, the method of spectral recording, selection, and processing of spectral variables is not very important. For the other variables, good models are obtained only with the appropriate choice of recording and processing of spectral data. Based on our results, we conclude that infrared spectroscopy offers a good supplemental or alternative method for the determination of vegetable oil quality.

## Figures and Tables

**Table 1:** Comparison of the *R_c_*^2^ and *R_v_*^2^ values obtained for models based on spectral and random data.

Spectral Preprocessing Method	*R*^2^ Type	Percent of Models with Good *R*^2^ Values						
		≥0.99	≥0.95	≥0.90	≥0.80	≥0.70	≥0.60	≥0.50
Raw spectral data	*R_c_*^2^ *R_v_*^2^	26%	56%	67%	73%	75%	78%	79%
		2%	7%	15%	23%	28%	32%	40%
First derivative of spectral data	*R_c_*^2^ *R_v_*^2^	37%	59%	68%	74%	77%	80%	84%
		1%	5%	12%	20%	23%	27%	30%
Second derivative of spectral data	*R_c_*^2^ *R_v_*^2^	39%	58%	66%	71%	78%	81%	85%
		0%	4%	9%	16%	18%	20%	23%
Normalized spectral data	*R_c_*^2^ *R_v_*^2^	38%	57%	68%	74%	76%	79%	83%
		4%	15%	18%	25%	29%	34%	39%
First derivative of normalized spectral data	*R_c_*^2^ *R_v_*^2^	47%	59%	67%	76%	79%	83%	86%
		6%	13%	17%	22%	26%	29%	33%
Second derivative of normalized spectral data	*R_c_*^2^ *R_v_*^2^	42%	58%	67%	73%	80%	84%	88%
		3%	8%	12%	17%	19%	22%	25%
SNV of spectral data	*R_c_*^2^ *R_v_*^2^	40%	60%	71%	75%	78%	81%	84%
		6%	16%	19%	24%	30%	35%	39%
First derivative of SNV spectral data	*R_c_*^2^ *R_v_*^2^	50%	63%	70%	78%	81%	84%	88%
		6%	13%	16%	21%	27%	30%	33%
Second derivative of SNV spectral data	*R_c_*^2^ *R_v_*^2^	45%	59%	68%	73%	80%	84%	87%
		3%	9%	12%	17%	19%	22%	25%
Wavelet approximate coefficients of spectral data	*R_c_*^2^ *R_v_*^2^	24%	54%	66%	71%	74%	77%	78%
		2%	8%	15%	22%	27%	31%	41%
First derivative of wavelet approximate spectral data	*R_c_*^2^ *R_v_*^2^	33%	60%	68%	73%	76%	80%	85%
		1%	6%	13%	20%	25%	30%	32%
Second derivative of wavelet approximate spectral data	*R_c_*^2^ *R_v_*^2^	30%	53%	61%	68%	74%	78%	83%
		0%	4%	13%	19%	21%	25%	27%
Wavelet detail coefficients of spectral data	*R_c_*^2^ *R_v_*^2^	33%	55%	67%	74%	77%	80%	85%
		0%	6%	11%	20%	23%	28%	30%
First derivative of wavelet detail spectral data	*R_c_*^2^ *R_v_*^2^	29%	48%	60%	67%	72%	77%	81%
		0%	4%	13%	17%	20%	23%	25%
Second derivative of wavelet detail spectral data	*R_c_*^2^ *R_v_*^2^	29%	44%	54%	63%	69%	75%	81%
		0%	3%	7%	15%	18%	21%	24%
Random data	*R_c_*^2^ *R_v_*^2^	46%	59%	73%	87%	89%	90%	92%
		0%	0%	0%	0%	0%	0%	0%

*R_c_*^2^—determination coefficient of calibration set; *R_v_*^2^*—*determination coefficient of validation set.

**Table 2:** Percent of models with *R_v_*^2^ and *R_c_*^2^ values that are both above 0.9 or 0.5 for individual predictive variables using different spectra recording techniques, resolutions, averaged or separate spectra, and variable selection techniques. Higher percentages are marked in bold.

Dependent Variable		ATR	TRANS	R2	R4	R8	ALL	AVG	STD	CORR	CHEM
Palmitic acid	*R*^2^ ≥ 0.9	0%	0%	0%	0%	0%	0%	0%	0%	0%	0%
	*R*^2^ ≥ 0.5	16%	**34%**	9%	17%	**23%**	**33%**	16%	14%	**18%**	17%
Linoleic acid	*R*^2^ ≥ 0.9	16%	**37%**	13%	18%	**22%**	**28%**	25%	15%	18%	**20%**
	*R*^2^ ≥ 0.5	34%	**50%**	21%	29%	**33%**	**42%**	41%	27%	27%	**30%**
α-Linolenic acid	*R*^2^ ≥ 0.9	30%	**48%**	19%	25%	**33%**	**40%**	38%	25%	25%	**27%**
	*R*^2^ ≥ 0.5	39%	**50%**	24%	31%	**33%**	**45%**	44%	29%	29%	**31%**
Oleic acid	*R*^2^ ≥ 0.9	0%	0%	0%	0%	0%	0%	0%	0%	0%	0%
	*R*^2^ ≥ 0.5	4%	**28%**	5%	13%	**14%**	**23%**	8%	11%	9%	**13%**
Elaidic acid	*R*^2^ ≥ 0.9	0%	0%	0%	0%	0%	0%	0%	0%	0%	0%
	*R*^2^ ≥ 0.5	**3%**	2%	1%	**3%**	1%	2%	2%	2%	2%	1%
Stearic acid	*R*^2^ ≥ 0.9	0%	**2%**	0%	1%	1%	**2%**	0%	1%	0%	1%
	*R*^2^ ≥ 0.5	14%	**25%**	9%	12%	**18%**	**31%**	8%	12%	13%	**15%**
Unsaponifiable matter	*R*^2^ ≥ 0.9	0%	0%	0%	0%	0%	0%	0%	0%	0%	0%
	*R*^2^ ≥ 0.5	2%	2%	1%	**2%**	1%	2%	2%	1%	1%	**2%**
Acid value	*R*^2^ ≥ 0.9	0%	0%	0%	0%	0%	0%	0%	0%	0%	0%
	*R*^2^ ≥ 0.5	0%	0%	0%	0%	0%	0%	0%	0%	0%	0%
Saponification value	*R*^2^ ≥ 0.9	0%	0%	0%	0%	0%	0%	0%	0%	0%	0%
	*R*^2^ ≥ 0.5	0%	0%	0%	0%	0%	0%	0%	0%	0%	0%
Ester value	*R*^2^ ≥ 0.9	0%	0%	0%	0%	0%	0%	0%	0%	0%	0%
	*R*^2^ ≥ 0.5	0%	0%	0%	0%	0%	0%	0%	0%	0%	0%
Hydroxyl value	*R*^2^ ≥ 0.9	0%	0%	0%	0%	0%	0%	0%	0%	0%	0%
	*R*^2^ ≥ 0.5	0%	0%	0%	0%	0%	0%	0%	0%	0%	0%
Iodine value	*R*^2^ ≥ 0.9	13%	**30%**	16%	10%	**17%**	16%	**26%**	14%	14%	14%
	*R*^2^ ≥ 0.5	41%	**50%**	26%	31%	**33%**	45%	45%	29%	30%	**31%**
Peroxide value	*R*^2^ ≥ 0.9	0%	0%	0%	0%	0%	0%	0%	0%	0%	0%
	*R*^2^ ≥ 0.5	0%	0%	0%	0%	0%	0%	0%	0%	0%	0%

*R*^2^—both determination coefficients; ATR—spectra collected with ATR technique; TRANS—spectra collected with transmissive technique; R2—resolution of spectra 2 cm^−1^; R4—resolution of spectra 4 cm^−1^; R8—resolution of spectra 8 cm^−1^; ALL—three separate spectra used for each sample; AVG—averaged spectra used for each sample; STD—standard deviation used for variable selection; CORR—correlation coefficient used for variable selection; CHEM—variable selected based on absorption of important chemical bonds.

**Table 3:** Three best models for each dependent variable are presented with the spectral measurement parameters, model building parameters, and evaluation parameters RMSECV, RMSEC, RMSEP, *R_c_*^2^, and *R_v_*^2^.

	ATR or Trans	Resolution	All or Averaged sp.	Variable Selection	Preprocessing	PLS Factors	RMSECV	RMSEC	RMSEP	Calibration Range	*R_c_* ^2^	*R_v_* ^2^
Palmitic acid	Trans	R2	Avg	STD	NOR	9	1.54	0.56	0.55	1.74–9.90	0.91	0.89
	Trans	R8	All	CORR	WD	12	0.66	0.42	0.56		0.95	0.89
	Trans	R2	Avg	STD	WA	11	1.60	0.44	0.59		0.95	0.87
Linoleic acid	Trans	R8	All	STD	NOR 1st D	16	2.99	1.30	1.23	6.63–80.00	0.99	1.00
	Trans	R8	All	CHEM	NOR 1st D	17	3.00	1.07	1.40		1.00	1.00
	Trans	R8	All	STD	SNV 1st D	16	2.53	1.13	1.47		1.00	0.99
α-Linolenic acid	Trans	R2	All	CORR	SNV	18	1.69	0.62	0.98	0.00–68.50	1.00	1.00
	ATR	R8	Avg	CHEM	SNV	7	2.74	0.90	1.19		1.00	1.00
	Trans	R2	All	CHEM	SNV	18	1.70	0.53	1.25		1.00	1.00
Oleic acid	Trans	R8	All	STD	NOR 1st D	14	1.97	1.19	3.75	7.28–34.50	0.97	0.75
	Trans	R4	All	CHEM	SNV 1st D	18	2.12	0.14	3.76		1.00	0.75
	Trans	R8	All	CHEM	NOR 1st D	14	1.97	1.17	3.83		0.97	0.74
Elaidic acid	Trans	R4	All	STD	1st D	13	0.73	0.24	0.48	0.00–4.25	0.96	0.72
	Trans	R4	All	STD	WD	13	0.72	0.27	0.48		0.95	0.72
	ATR	R4	Avg	CHEM	NOR	3	1.16	0.85	0.50		0.54	0.69
Stearic acid	Trans	R4	All	CHEM	NOR 1st D	10	0.99	0.49	0.39	0.00–5.98	0.89	0.92
	Trans	R8	All	STD	WA	20	0.67	0.34	0.40		0.94	0.92
	Trans	R4	All	STD	NOR	17	0.72	0.37	0.41		0.94	0.92
Unsaponifiable matter	ATR	R2	Avg	CHEM	RAW	5	0.41	0.17	0.12	0.33–2.20	0.85	0.84
	ATR	R2	Avg	CHEM	WA	18	0.40	0.00	0.16		1.00	0.73
	Trans	R8	Avg	CORR	NOR 2nd D	20	0.44	0.00	0.18		1.00	0.65
Acid value	Trans	R2	Avg	CHEM	2nd D	9	1.48	0.05	0.55	0.112–11.2	1.00	−0.98
	Trans	R2	Avg	CORR	NOR 2nd D	7	1.20	0.18	0.56		0.99	−1.07
	Trans	R4	Avg	CORR	WD 2nd D	5	1.30	0.67	0.57		0.91	−1.09
Saponification value	ATR	R4	Avg	CORR	WA	1	3.63	2.65	0.72	178–196	0.23	0.26
	ATR	R2	Avg	CORR	WA	1	3.63	2.63	0.72		0.24	0.25
	ATR	R4	Avg	CORR	RAW	1	3.62	2.65	0.72		0.23	0.25
Ester value	ATR	R8	Avg	CHEM	NOR 2nd D	10	3.47	0.02	0.86	176–194	1.00	0.22
	Trans	R4	Avg	STD	RAW	1	3.74	3.47	0.95		0.11	0.04
	Trans	R4	Avg	STD	WA	1	3.74	3.47	0.95		0.11	0.04
Hydroxyl value	Trans	R4	Avg	CHEM	NOR	1	3.16	2.72	1.31	2.70–19.4	0.19	0.26
	Trans	R4	Avg	CORR	WD	1	3.07	2.52	1.32		0.30	0.25
	Trans	R4	Avg	CORR	1st D	1	3.05	2.54	1.33		0.29	0.23
Iodine value	ATR	R8	Avg	STD	NOR 2nd D	2	7.73	4.47	1.94	99–204	0.96	0.98
	ATR	R8	Avg	CORR	NOR	1	6.06	5.66	2.18		0.93	0.98
	ATR	R8	Avg	CORR	NOR 1st D	2	6.91	5.26	2.36		0.94	0.97
Peroxide value	ATR	R8	Avg	CORR	NOR	1	21.36	18.38	18.87	9.29–123	0.39	0.49
	Trans	R8	All	CHEM	1st D	20	11.32	2.01	18.92		0.99	0.49
	Trans	R8	All	CHEM	WA 2nd D	18	11.14	3.54	19.03		0.98	0.49

*R_c_*^2^*—*determination coefficient of calibration set; *R_v_*^2^*—*determination coefficient of validation set; RMSECV—root mean error of cross-validation (for PLS); RMSEC—root-mean-square error of calibration; RMSEP—root-mean-square error of prediction; ATR—spectra collected with ATR technique; TRANS—spectra collected with transmissive technique; R2—resolution of spectra 2 cm^−1^; R4—resolution of spectra 4 cm^−1^; R8—resolution of spectra 8 cm^−1^; All three separate spectra used for each sample; Avg—averaged spectra used for each sample; STD—standard deviation used for variable selection; CORR—correlation coefficient used for variable selection; CHEM—variable selected based on absorption of important chemical bonds; 1st D—first derivative; 2nd D—second derivative; NOR—normalized spectra; SNV—spectra normalized with standard normal variate; WA—approximate wavelet coefficients of spectra; WD—detailed wavelet coefficients of spectra.

**Table 4:** Comparison of the FT-IR method and currently used methods for vegetable oil characterization.

	Titration (Acid, Hydroxyl, Iodine, Peroxide, Saponification Value)	Gas Chromatography (Fatty Acid Content)	FT-IR
Method development	developed	developed	has to be developed and validated
Time consumption	long	long	fast
Amount of sample	grams	miligrams	miligrams
Repeatability	poor	good	good
Chemicals	toxic organic	toxic organic	none
Ease of analysis	good laboratory skills	good laboratory skills	easy
Laboratory equipment	basic	expensive	expensive

**Table 5:** Samples of vegetable oils used in the analysis.

Plant of the Oil Source	Latin Name of the Plant	Supplier	Calibration or Validation Dataset
Cranberry seed oil	*Vaccinum macrocarpon*	Behawe Naturprodukte, Germany	Calibration
Cranberry seed oil	*Vaccinum macrocarpon*	Alexmo Cosmetics, Germany	Calibration
Cranberry seed oil	*Vaccinum macrocarpon*	Dragonspice Naturwaren, Germany	Validation
Elderberry seed oil	*Sambucus nigra*	Baccararose, Germany	Calibration
Elderberry seed oil	*Sambucus nigra*	Behawe Naturprodukte, Germany	Calibration
Borage seed oil	*Borago officinalis*	Dragonspice Naturwaren, Germany	Validation
Borage seed oil	*Borago officinalis*	Tovarna Organika, Slovenia	Validation
Borage seed oil	*Borago officinalis*	Caelo, Germany	Calibration
Borage seed oil	*Borago officinalis*	Farmalabor, Italy	Calibration
Blackcurrant seed oil	*Ribes nigrum*	Dragonspice Naturwaren, Germany	Calibration
Blackcurrant seed oil	*Ribes nigrum*	Behawe Naturprodukte, Germany	Calibration
Hemp seed oil	*Cannabis sativa*	Dragonspice Naturwaren, Germany	Calibration
Hemp seed oil	*Cannabis sativa*	Tovarna Organika, Slovenia	Calibration
Hemp seed oil	*Cannabis sativa*	Manske, Germany	Validation
Raspberry seed oil	*Rubus idaeus*	Tovarna Organika, Slovenia	Validation
Raspberry seed oil	*Rubus idaeus*	Dragonspice Naturwaren, Germany	Calibration
Raspberry seed oil	*Rubus idaeus*	Behawe Naturprodukte, Germany	Calibration
Black mustard seed oil	*Brassica nigra*	Behawe Naturprodukte, Germany	Calibration
Walnut seed oil	*Juglans regia*	Baccararose, Germany	Calibration
Walnut seed oil	*Juglans regia*	Caelo, Germany	Calibration
Sea buckthorn seed oil	*Hippophae rhamnoides*	Dragonspice Naturwaren, Germany	Calibration
Sea buckthorn seed oil	*Hippophae rhamnoides*	Behawe Naturprodukte, Germany	Calibration
Evening primrose seed oil	*Oenothera biennis*	Dragonspice Naturwaren, Germany	Validation
Evening primrose seed oil	*Oenothera biennis*	Farmalabor, Italy	Calibration
Evening primrose seed oil	*Oenothera biennis*	Alexmo Cosmetics, Germany	Calibration
Evening primrose seed oil	*Oenothera biennis*	Caelo, Germany	Validation
Rosehip seed oil	*Rosa canina*	Manske, Germany	Calibration
Rosehip seed oil	*Rosa canina*	Alexmo Cosmetics, Germany	Calibration
Chia seed oil	*Salvia hispanica*	Baccararose, Germany	Calibration
Chia seed oil	*Salvia hispanica*	Dragonspice Naturwaren, Germany	Calibration
Perilla seed oil	*Perilla frutescens*	Baccararose, Germany	Calibration
Black cumin seed oil	*Nigella sativa*	Caelo, Germany	Calibration
Sacha inchi seed oil	*Plukenetia volubilis*	Magnolija, Slovenia	Calibration
Kiwi seed oil	*Actinidia chinensis*	Dragonspice Naturwaren, Germany	Calibration
Lineseed oil	*Linum usitatissimum*	Baccararose, Germany	Validation
Lineseed oil	*Linum usitatissimum*	Farmalabor, Italy	Calibration
Lineseed oil	*Linum usitatissimum*	Caelo, Germany	Calibration

## Data Availability

The data presented in this study are available in supplementary material.

## Supplementary Materials

The following supporting information can be downloaded at: https://www.mdpi.com/article/10.3390/molecules27103190/s1, Figure S1: Average ATR spectra of borage seed oil and evening primrose seed oil samples; Figure S2: Average transmissive spectra of borage seed oil and evening primrose seed oil samples; Table S1: Percent of models with *R_v_^2^* and *R_c_^2^* values that are both higher than 0.9 or 0.5 for different predictive variables and different resolutions using different preprocessing methods

## Sample Availability

Samples of the compounds are not available.
