# Supplementary material for: Statistical FT-IR Spectroscopy for the Characterization of 17 Vegetable Oils
Source: Molecules. 2022 May 17;27(10):3190. doi: 10.3390/molecules27103190 (PMC9147165; doi:10.3390/molecules27103190)
Supplement: Supplementary file 1 [file molecules-27-03190-s001.zip › molecules-1713348-supplementary.pdf]

Supplementary Materials

Table S1: Percent of models with R_v^2 and R_c^2 values that are both higher than 0.9 or 0.5 for different predictive variables and different resolutions using different preprocessing methods.

**Dependent variable**		**Raw spectra**	**1st Derivative**	**2nd Derivative**	**Normalization**	**NOR 1st Der**	**NOR 2nd Der**	**SNV**	**SNV 1st Der**	**SNV 2nd Der**	**WA**	**WA 1st Der**	**WA 2nd Der**	**WD**	**WD 1st Der**	**WD 2nd Der**
Palmitic	$R^2 \geq 0.9$	0	0	0	0	0	0	0	0	0	0	0	0	0	0	0
acid	$R^2 \geq 0.5$	53	53	28	69	61	39	72	64	36	56	53	47	47	33	25
Linoleic	$R^2 \geq 0.9$	64	53	25	78	64	39	92	64	39	75	50	47	44	39	17
acid	$R^2 \geq 0.5$	100	78	72	100	83	72	100	83	72	100	89	75	81	75	72
α-linolen	$R^2 \geq 0.9$	92	72	64	100	72	67	100	72	67	89	89	75	75	75	53
acid	$R^2 \geq 0.5$	100	89	75	100	89	75	100	89	75	100	100	86	94	83	78
Oleic acid	$R^2 \geq 0.9$	0	0	0	0	0	0	0	0	0	0	0	0	0	0	0
	$R^2 \geq 0.5$	39	22	17	42	36	25	53	39	25	42	36	25	28	25	22
Elaidic	$R^2 \geq 0.9$	0	0	0	0	0	0	0	0	0	0	0	0	0	0	0
acid	$R^2 \geq 0.5$	3	6	6	19	6	3	6	6	3	3	3	0	6	0	0
Stearic	$R^2 \geq 0.9$	6	0	0	3	8	0	0	6	0	6	0	0	0	0	0
acid	$R^2 \geq 0.5$	58	36	28	64	39	28	58	31	28	56	39	31	39	25	31
Unsaponi	$R^2 \geq 0.9$	0	0	0	0	0	0	0	0	0	0	0	0	0	0	0
matter	$R^2 \geq 0.5$	8	8	0	8	3	3	11	3	3	8	3	3	3	0	0
Acid value	$R^2 \geq 0.9$	0	0	0	0	0	0	0	0	0	0	0	0	0	0	0
	$R^2 \geq 0.5$	0	0	0	0	0	0	0	0	0	0	0	0	0	0	0
Saponific	$R^2 \geq 0.9$	0	0	0	0	0	0	0	0	0	0	0	0	0	0	0
value	$R^2 \geq 0.5$	0	0	0	0	0	0	0	0	0	0	0	0	0	0	0
Ester	$R^2 \geq 0.9$	0	0	0	0	0	0	0	0	0	0	0	0	0	0	0
value	$R^2 \geq 0.5$	0	0	0	0	0	0	0	0	0	0	0	0	0	0	0
Hydroxy	$R^2 \geq 0.9$	0	0	0	0	0	0	0	0	0	0	0	0	0	0	0
value	$R^2 \geq 0.5$	0	0	0	0	0	0	0	0	0	0	0	0	0	0	0
Iodine	$R^2 \geq 0.9$	28	31	22	53	72	44	53	67	56	28	36	44	28	56	25
value	$R^2 \geq 0.5$	100	97	72	100	100	75	100	100	75	100	100	89	94	83	75
Peroxide	$R^2 \geq 0.9$	0	0	0	0	0	0	0	0	0	0	0	0	0	0	0
value	$R^2 \geq 0.5$	0	0	0	0	0	0	0	0	0	0	0	0	0	0	0
Resolution																
2 cm^{-1}	$R^2 \geq 0.9$	14	12	5	18	13	6	21	12	6	14	12	10	10	10	1
	$R^2 \geq 0.5$	35	20	13	37	23	12	39	23	13	36	27	16	21	12	12
4 cm^{-1}	$R^2 \geq 0.9$	12	10	8	16	15	10	17	15	12	13	13	13	11	13	8
	$R^2 \geq 0.5$	35	35	22	42	37	25	39	37	25	35	33	31	33	28	21
8 cm^{-1}	$R^2 \geq 0.9$	17	15	13	20	22	19	19	21	19	18	15	15	13	16	13
	$R^2 \geq 0.5$	37	35	33	37	37	37	37	36	35	37	37	35	37	36	37

R^2 – both determination coefficients; 1st Der – first derivative; 2nd Der – second derivative; NOR – normalized spectra; SNV – spectra normalized with standard normal variate; WA – approximate wavelet coefficients of spectra; WD – detailed wavelet coefficients of spectra.

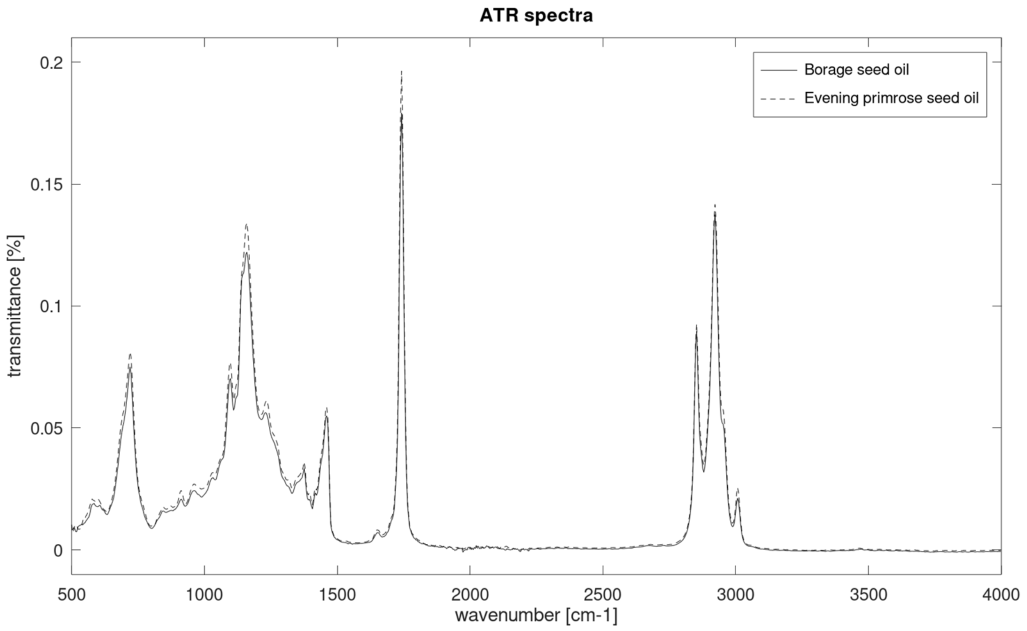

Figure S1: Average ATR spectra of borage seed oil and evening primrose seed oil samples.

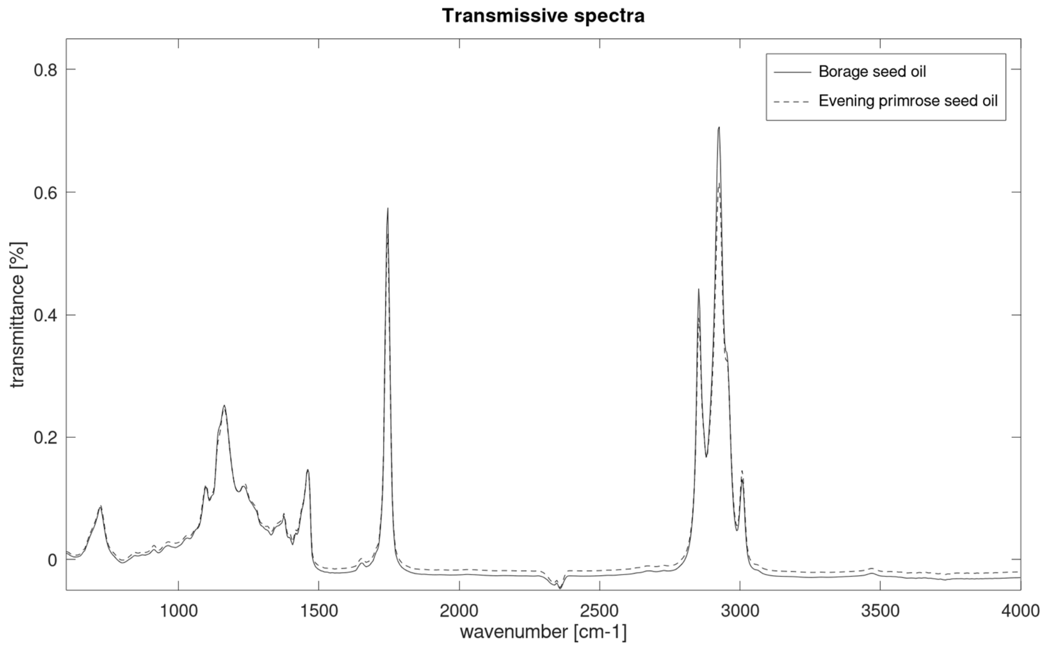

Figure S2: Average transmissive spectra of borage seed oil and evening primrose seed oil samples.
